# Post-approval safety studies of vaccines in pregnancy: available regulatory guidance and next steps towards the more efficient generation of safety evidence

**DOI:** 10.3389/fdsfr.2025.1648854

**Published:** 2025-11-26

**Authors:** Sarah C. MacDonald, Adrienne P. Guignard, Flor M. Munoz, Eileen O. Dareng, Kourtney J. Davis, Marina Amaral de Avila Machado, Shahar Shmuel, Laura Taddei, Lydie Marcelon

**Affiliations:** 1 Safety Surveillance Research, Worldwide Medical and Safety, Pfizer, Inc., Ottawa, ON, Canada; 2 Global Epidemiology, Organization of the Chief Medical Officer, GlaxoSmithKline, Wavre, Belgium; 3 Departments of Pediatrics and Molecular Virology and Microbiology, Baylor College of Medicine, Houston, TX, United States; 4 Safety Epidemiology and Risk Management, Global Patient Safety, AstraZeneca, Cambridge, United Kingdom; 5 Global Epidemiology, Johnson & Johnson, Horsham, PA, United States; 6 Epidemiology & Benefit-Risk, Sanofi, Toronto, ON, Canada; 7 Safety Surveillance Research, Worldwide Medical and Safety, Pfizer, Inc., New York, NY, United States; 8 Global Epidemiology, GlaxoSmithKline (GSK), Siena, Italy; 9 Epidemiology & Benefit-Risk, Sanofi, Lyon, France

**Keywords:** pregnancy, vaccines, safety, regulatory guidance, post-approval studies, maternal immunization, pharmacovigilance, pregnancy registries

## Abstract

**Background:**

The “Beyond COVID-19 Monitoring Excellence” (BeCOME) initiative was established to leverage the successful multi-stakeholder collaborations achieved during the COVID-19 pandemic to increase efficiencies for future pandemic preparedness. At the first BeCOME cross-stakeholder meeting, the following key challenge was identified: Inconsistent guidance from regulatory authorities on the conduct of post-marketing safety studies in pregnant people.

**Objectives:**

This article aims to describe examples of post-approval safety studies sponsored by marketing authorization holders (MAHs) for vaccines in pregnancy, analyze existing regulatory guidelines for these studies, and identify areas warranting more detailed guidance.

**Example Studies:**

Fifteen vaccine post-approval safety studies in pregnant people with publicly available methodology were identified from the European Union (EU) Post-authorisation Study (PAS) Register and supplemented by a literature search. Studies were selected to cover both primary data collection and secondary use of data, as well as for vaccines recommended and not recommended during pregnancy. The identified studies varied in their type (active vs. passive surveillance), comparators, outcomes, exposure windows, target sample sizes, and study durations.

**Existing Guidance:**

Five applicable guidance documents were identified from two health authorities [the United States (US) Food and Drug Administration (FDA) and the European Medicines Agency (EMA)], ranging in publication date from 2005–2023. Available guidance from both agencies included robust discussions of comparator groups, outcomes, and exposure periods. However, vaccine-specific recommendations were notably lacking.

**Actionable Recommendations:**

Additional vaccine-specific guidance is needed across regulatory authorities. Key areas of focus should include: the selection of study design by vaccine type, appropriate comparators for vaccine research, vaccine-specific recommendations for ascertaining exposure, a harmonized and prioritized list of outcomes, greater understanding of the role of outcome validation, pre-defined target minimal detectable risks by priority outcomes, appropriate durations of follow-up, additional guidance regarding the implementation of multi-stakeholder collaborations, and a framework for the use of rapid cycle analyses.

**Conclusion:**

There is wide variation in study designs and approaches for assessing vaccine safety in pregnancy post-approval, influenced by differences in vaccines, target populations, sponsors, and study periods. Harmonizing regulatory guidance and standards will enhance the consistency of data collection, as well as the comparability and validity of study conclusions.

## 1 Introduction

The “Beyond COVID-19 Monitoring Excellence” (BeCOME) initiative was established in 2022 with the goal of leveraging the successful multi-stakeholder collaborations achieved during the COVID-19 pandemic to continue to increase efficiencies for future pandemic preparedness. The first cross-stakeholder meeting of BeCOME was held on June 11–13, 2023 (Bauchau et al., 2025). This meeting aimed to identify and make recommendations on strategic priorities, solutions, timelines, and models for collaboration to expand on the cooperation across organizations (pharmaceutical, academic, government, etc.) initiated during the COVID-19 pandemic. The following key challenge to studying vaccine exposure and safety in pregnant populations was identified: “Inconsistent guidance from regulatory authorities on the conduct of post-marketing safety studies in pregnant people that leads to inefficiency in study design, feasibility assessments, and, in turn, later delivery of actionable evidence to inform safety decision making.”

Indeed, the current mechanism of generating post-approval evidence of medication and vaccine safety during pregnancy and among breastfeeding people appears inefficient with minimal impact. For example, a landscape analysis of observational studies focusing on the safety evaluation of medicines and vaccines used during pregnancy and breastfeeding identified that, of 46 completed studies, only two resulted in a product label update (lamotrigine, oseltamivir) (Roque et al., 2022). In addition to their relatively limited impact, these post-approval safety studies are considerably resource intensive. For example, the same landscape review observed that the studies conducted using primary data collection [defined as data actively collected for the purpose of the study (e.g., through surveys, interviews, or observation)] averaged 7.5 years to completion, while the secondary database studies [defined as the use of already existing data (e.g., from medical records or health insurance claims)] averaged 2.9 years.

A lack of harmonization in global regulations on post-approval safety research for medicines and vaccines in pregnant and breastfeeding people likely contributes to inefficiencies in study conduct across regions. Indeed, in contrast with available guidelines for pre-approval clinical trials, a recent global review of existing regulations and guidance for post-approval safety studies in pregnancy identified very few countries with available guidance (Alexe et al., 2024a; Alexe et al., 2024b). In the guidance that does exist [from the United States [US] Food and Drug Administration [FDA] or the European Medicines Agency (EMA)], inconsistent requirements can make it difficult for marketing authorization holders (MAHs) to know how studies should be designed and may result in the duplication of efforts across studies and regions. While many low- and middle-income countries (LMICs) do not have their own specific guidance for post-approval safety studies in pregnancy, these countries may often adopt or reference FDA and EMA guidance as regulatory frameworks. Gaps in existing guidance are also present, particularly for vaccine studies, leaving MAHs with limited information on expectations.

The objectives of this project were therefore to describe examples of post-approval safety studies sponsored by MAHs for vaccines in pregnancy, in order to provide a high-level overview of existing study designs and approaches, to analyze existing regulatory guidelines for post-approval safety studies in pregnancy, and to identify study design elements warranting more detailed guidance for vaccine research.

## 2 Overview of existing regulatory guidance for post-approval safety studies in pregnant populations

Current regulatory guidance for conducting post-approval non-interventional safety studies in pregnancy consists of the following documents from the US and the European Union (EU):^1^

- US Food and Drug Administration (FDA). Post approval pregnancy safety studies–Guidance for industry [Draft Guidance]. May 2019 (United States Food and Drug Administration, 2019).
- European Medicines Agency (EMA). Guideline on good pharmacovigilance practices (GVP) – Product- or population-specific considerations III: Pregnant and breastfeeding women [Draft Guideline]. December 2019 (European Medicines Agency, 2019).
- The European Network of Centres for Pharmacoepidemiology and Pharmacovigilance (ENCePP). Annex 2 to the Guide on Methodological Standards in Pharmacoepidemiology (Revision 11). July 2023 (The European Network of Centres for Pharmacoepidemiology and Pharmacovigilance, 2010).
- EMA. Guideline on the exposure to Medicinal Products during pregnancy. November 2005 (European Medicines Agency, 2005).

A comparison of the guidelines is presented in Table 1, and discussed in more detail in the sections below.

**TABLE 1:** Overview of regulatory guidance for post-approval safety studies in pregnancy.

Topic	Summary of US FDA guidance (United States Food and Drug Administration, 2019)	Summary of EMA guidance (European Medicines Agency, 2019; The European Network of Centres for Pharmacoepidemiology and Pharmacovigilance, 2010; European Medicines Agency, 2005)
Study population	**Specific to Primary Data Collection:** • Strive to enroll participants prospectively before the conduct of any prenatal tests, however do not exclude those enrolled retrospectively • Marketing authorization holders (MAHs) are encouraged to work together directly or through consortia to develop or support multi-product registries**Specific to Secondary Data Analysis:** • Strengths and limitations of the following data source types are highlighted: electronic health data (EHR), population-based surveillance and national registries/registers (e.g., state-based surveillance, VAMPSS, ICBDSR), population-based case control studies • Identifying eligible live birth pregnancies is typically straightforward based on reliable codes in EHR data • Under ascertainment of pregnancies ending in non-live birth outcomes could limit generalizability and result in bias (e.g., missing fatal birth defects) • Linkage to offspring records is recommended	**Specific to Primary Data Collection:** • Prospective enrollment of participants, ideally following from pre-conception to end of pregnancy with follow-up of live infants to evaluate post-natal outcomes building upon existing infrastructure, is preferred; retrospective enrollment should not be discouraged, and all outcomes should be included in the report • Recommend the use of existing disease-specific registries over product-specific registries **Specific to Secondary Data Analysis:** • Potential data sources available include regional or nationwide population-based medical databases, prescription databases, general practice databases, birth cohorts, congenital malformation registries, product-or disease specific pregnancy registries and exposure cohorts through teratology information services • Recommend using multiple databases from different countries that may be combined via meta-analysis techniques to overcome sample size concerns • Considerations for selection of a data source and/or common data model (CDM) fit for purpose include: (1) adaptability to a specific question, (2) transparency to reproduce findings, (3) ease and speed of use, (4) adequate capture of exposure and outcomes, (5) ability to link mother and child, (6) capture of pregnancy specific information (e.g., gestational age, birthweight, delivery data), (7) sufficient duration of follow-up in the child • Summaries of algorithms used in different data sources to identify pregnancies are provided • Data sources should be able to link mothers’ and infants’ records • Validation of algorithms to identify pregnancies and/or validation of linkages of secondary data may be recommended
Comparator selection	• Recommend a concurrent internal comparator with the same disease (and disease severity, if possible) that is unexposed to the product of interest • If using population-based surveillance systems (e.g., MACDP), the limitations of these systems should be considered • External databases with background rates from the population with the disease may be useful • Recommend the use of multiple comparator groups when feasible • If using a case-control design, controls should be selected from the same disease population as the cases **Specific to Primary Data Collection:** • A cohort exposed to different treatment regimens (besides the product of interest) within the same multiproduct or disease-based registry study may serve as additional internal comparators. This is an advantage of using multiproduct registries	• Use of different comparators, sibling designs, self-controlled designs, and positive and negative controls can help to reduce bias • Studies should explicitly address and justify whether different exposures will be combined (e.g., drugs within the same pharmacological class) **Specific to Primary Data Collection:** • Disease-based registries are generally more informative than product-specific ones, as they enable longitudinal assessment of treatment patterns, product comparisons, and outcomes in unexposed populations
Exposure definition	• Collect detailed information on start/stop dates, dose, frequency, duration, and indication for all products taken throughout pregnancy and in the period before pregnancy • Ensure timing of exposures relative to gestational age is accurate • Recommend stratifying analyses by gestational timing of exposure (evaluate first trimester exposures separately for major congenital malformations [MCMs]) **Specific to Primary Data Collection:** • Prospective data collection is recognized as a means to reduce exposure misclassification and recall bias by capturing detailed, time-specific exposure information before pregnancy outcomes are known **Specific to Secondary Data Analysis:** • Accurate gestational age estimates are critical for determining exposure timing during pregnancy but specific codes may not recorded. Methods used to estimate gestational age are outlined • When using birth defect registry information, MAHs may need to supplement the registries with drug or biological product exposure information via targeted maternal interviews and/or linkage to prescription information. Strengths and limitations of maternal interviews and approach to minimize recall bias are highlighted • For population-based case-control studies, medical record review to collect additional exposure data is recommended	• Information on timing and dose should be recorded as accurately as possible • Specifically address and justify how exposure windows will be studied, recognizing that accurate measurement of medicine uses during embryo-fetal development (i.e., the relevant risk window) requires precise determination of both pregnancy onset and timing of drug exposure • Exposure ascertainment should stop once the at-risk period of the outcome ends. Susceptible gestational age periods by outcome are described in the guidance • Consider misclassification errors that may result from incomplete recording of diagnoses or exposure (e.g., recall bias) • Sensitivity analyses should be conducted to address potential misclassification **Specific to Secondary Data Analysis:** • Supplementary information (e.g., from surveys or interviews in a subset of the population) can help to inform treatment adherence and sensitivity analyses
Selection of outcomes	• Recommend including both live birth and non-live birth outcomes • Appendix A of the guidance includes a recommended list of data elements (including outcomes) to be included when designing a registry. It is noted that these outcomes are also applicable to complementary studies (e.g., studies using secondary databases)	• Study should explicitly address and justify which outcomes will be evaluated in the pregnancy and the child • MCMs are often studied • Ability to evaluate the health and (neuro)developmental outcomes in the child is of special interest • Long-term follow-up of childhood outcomes may be required depending on biological plausibility and other available data • Commonly studied pregnancy outcomes are listed in Table 1 of the ENCePP guidance (The European Network of Centres for Pharmacoepidemiology and Pharmacovigilance, 2010) **Specific to Primary Data Collection:** • Information to be collected when establishing a pregnancy exposure questionnaire are listed in Appendix 1 of the GVP guidance (European Medicines Agency, 2019)
Definition of MCMs	• A standardized classification system should be utilized • Criteria should be clearly stated that define which abnormalities will be excluded and which will be defined as “major defects” **Specific to Secondary Data Analysis:** • MCMs should not be defined using single diagnostic codes (i.e., could reflect coding errors, rule-out diagnoses, etc.) • Validity of diagnostic codes for specific birth defects varies greatly by the birth defect and data source	• MCMs are often defined using the European Registration of Congenital Anomalies and Twins Network (EUROCAT) methodology • Assessment of MCMs as a composite, heterogeneous outcome has substantial limitations (i.e., will be biased towards the null and can lead to a false sense of security) • Since the prevalence of specific birth defects is low, it may not be feasible to study individual birth defects in most studies • Congenital malformations should be ascertained at birth and throughout the first year of life and beyond
Validation of outcomes	**Specific to Primary Data Collection:** • All reported MCM cases should be reviewed and classified by an expert clinical geneticist or dysmorphologist • Reviewer and method of assessment should be consistent across groups and blinded to exposure status **Specific to Secondary Data Analysis:** • All outcomes should be validated [using a gold-standard method (e.g., medical chart)] unless using a high-performing algorithm that has been previously validated in the same (or similar) database • MCM outcomes should be validated by clinical experts and/or should be ascertained via linkage to birth defect registries or birth certificate data	No corresponding guidance
Covariates and potential confounders	• Clinical severity measures should be collected to reduce the potential for confounding by indication • The guidance lists the following other potential confounders that are recommended to be collected: socioeconomic status, maternal age, tobacco exposure, alcohol use, recreational substance use, maternal body mass index (BMI), folic acid and vitamin use during pregnancy, obstetrical history, medical history, family history of adverse pregnancy outcomes and others (Caton, 2012)	• Family history is an important source of bias; it tends to be recorded selectively once a child with a malformation is born • Severity of disease and indication should be accounted for • Impact of potential confounding may be assessed via triangulation • Potential confounders include lifestyle factors (e.g., smoking, alcohol intake, folic acid intake, BMI) or other factors relating to fetal or neonatal development (e.g., maternal pregnancy complication, prior history of negative pregnancy outcomes or pre-term birth, prescription of known teratogenic or fetotoxic medicines, maternal disease likely to cause fetal or neonatal adverse consequences) • Information to be collected when establishing a pregnancy exposure questionnaire are listed in Appendix 1 of the GVP guidance (European Medicines Agency, 2019)
Sample size	• Target sample size should be presented in a statistical analysis plan (SAP) and be based on (1) feasibility in the patient population (2) and power calculations using the outcome of interest with the lowest background rate • Choice of background rates should be justified	• Sample size should be sufficient to assess individual or small groups of birth defects (rather than one composite MCM endpoint) and maintain a low likelihood of Type I and II errors **Specific to Secondary Data Collection:** • Recommend using multiple databases from different countries that may be combined via meta-analysis techniques to overcome sample size concerns
Recruitment plans	**Specific to Primary Data Collection:** • Collaboration with external entities (e.g., existing registries, medical societies, patient advocacy groups) is encouraged to increase awareness of the registry and its purpose to generate evidence • Recruitment strategies can be facility-based, healthcare provider (HCP)-initiated, or patient-initiated • Successful strategies to encourage participation include incentives, and the employment of empathetic, culturally sensitive, and personable study staff	No corresponding guidance
Retention plans	**Specific to Primary Data Collection:** • Retention plans should be developed and should include ways to maintain participation in the registry, follow-up with participants over time, and track retention • HCP retention should also be considered, Strategies may include reducing the burden of data collection, and providing access to results (including data from interim reports) • To improve patient retention: communicate directly with the patient, emphasize the registry mission, share study results, establish and maintain a longitudinal relationship between interviewer and participant, collect contact information of family members/friends, and have a flexible follow-up schedule	No corresponding guidance
Data collection and Follow-up	• Appendix A in guidance has a list of recommended data elements. It is noted that these elements are also applicable to complementary studies (e.g., studies using secondary databases) **Specific to Primary Data Collection:** • Ensure identical data collection efforts between exposed and comparator study groups • Avoid sole reliance on prenatal HCP records (e.g., infant HCP may be best resource for infant-related outcomes) • Include MCMs ascertained from postmortem examination of non-live birth fetuses • Include a plan for follow-up contacts during/after pregnancy	• Data to be collected includes maternal, neonatal and fetal information • Long-term follow-up of childhood outcomes may be required depending on biological plausibility and other available data **Specific to Primary Data Collection:** • Information to be collected when establishing a pregnancy exposure questionnaire are listed in Appendix 1 of the GVP guidance (European Medicines Agency, 2019)
Data analysis and presentation	• The primary study outcome should be assessed among pregnancies resulting in each of: live birth, miscarriage, elective termination or fetal death/stillbirth • Case validation should be performed • SAP should specify methods for characterizing risk and evaluating hypotheses regarding potential association between exposure and outcomes • Choice of format for data presentation will be study-specific • Separate analyses should be performed for each outcome of interest, stratified by gestational timing of exposure	• Unit of analysis should be specified due to the possibility of multiple pregnancies and account for potential clustering (e.g., through multilevel models, sibling comparison design) • Examples for addressing common issues and biases: o Confounding by indication: compare patient characteristics in exposure groups and perform sensitivity analyses. Assess impact by triangulation o Left truncation and conditioning on pregnancy outcomes o Immortal time bias o Misclassification: sensitivity analyses should be conducted o Competing endpoints
Independent data monitoring committee/scientific advisory board	**Specific to Primary Data Collection:** • Registries are encouraged to have an independent data monitoring committee/scientific advisory board that reviews the data, classifies outcomes (including MCMs), and assists with results dissemination • Role of the committee should be outlined in the study protocol	No corresponding guidance
Multiproduct pregnancy registries	**Specific to Primary Data Collection:** • MAHs are encouraged to work together directly or through consortia to develop or support multi-product registries	• The use of existing registries or databases are preferred to enhance efficiency in follow-up, comparators, and infrastructure **Specific to Primary Data Collection:** • Recommend the use of existing disease-specific registries over product-specific registries
Discontinuation	**Specific to Primary Data Collection:** • Criteria for termination of a registry: (1) the scientific objectives have been met, (2) infeasible to collect sufficient information, or (3) other preferrable methods have been identified to capture the relevant information	No corresponding guidance
Population-Based surveillance of birth defects	• Described as an alternative data source to collect information about birth defects • Key strengths include large population-based sample sizes, adjudication of birth defects, and standardized use of birth defect definitions • However, some international birth defect registries may include MCMs associated with chromosomal abnormalities, and may have limited information about non-live birth outcomes • When using birth defect registry information, MAHs may need to supplement the registries with drug or biological product exposure information via targeted maternal interviews and/or linkage to prescription information	• For improved efficiency, recommend a hybrid study design using product-specific information and available public data sources (e.g., birth defect registries) • Data from the European Network of Teratology Information Services (ENTIS) may overestimate the drug-malformation association and should not be used in place of a formal epidemiologic study
Checklists	No corresponding guidance	• Checklists (e.g., ENCePP, and list of data collection elements) are helpful to promote researchers to give thought to each key element

Abbreviations: BMI, body mass index; CDM, common data model; EMA, european medicines agency; ENCePP, european network of centres for pharmacoepidemiology and pharmacovigilance; ENTIS, european network of teratology information services; EUROCAT, european registration of congenital anomalies and twins network; FDA, food and drug administration; ICBDSR, international clearinghouse for birth defects surveillance and research; GVP, Good Pharmacogivilance Practices; HCP, healthcare provider; MACDP, the Metropolitan Atlanta Congenital Defects Program; MAH(s) = marketing authorization holder(s); MCM(s) = major congenital malformation(s); SAP, statistical analysis plan; US, united states; VAMPSS, vaccines and medications in pregnancy surveillance system.

Guidance from the Brighton Collaboration (Munoz et al., 2015; Jones C. E., et al., 2016), while referenced in some sections of the manuscript, is not summarized in this table because this guidance is not specific to non-interventional studies, nor does originate from a regulatory authority.

## 3 Overview and examples of existing studies

When studying vaccine safety in pregnancy, recommended populations are a key determinant for identifying the safety evaluation approach. Vaccine exposure during pregnancy can be either (1) inadvertent, meaning the vaccine is not broadly recommended by vaccine advisory committees^2^ for use in pregnancy, however exposures among pregnant individuals may occur; or (2) intentional, meaning the vaccine is recommended for use in pregnancy by at least one vaccine advisory committee or it is deemed appropriate for use in pregnancy based on the benefit-risk profile for a pregnant individual.

Two broad aims can be achieved through intentional maternal immunization: (1) Pregnant people can be protected against infectious diseases that result in severe outcomes during pregnancy and (2) their newborn infants are protected by high titres of antigen-specific immunoglobulin G (IgG) antibody against diseases of particular importance in the newborn [e.g., tetanus, pertussis, respiratory syncytial virus [RSV], influenza and coronavirus disease 2019 (COVID-19)]. Generally, vaccines with intentional pregnancy exposure will accrue a larger sample size of exposed individuals in clinical and/or observational post-approval studies and, consequently, allow for a more robust assessment of safety outcomes than vaccines for which inadvertent exposure only may occur.

Overall, three main study types are commonly conducted to evaluate the safety of vaccines in pregnant populations. Their key features are summarized in Table 2. A pregnancy registry is a prospective cohort study design that actively enrolls pregnant persons to systematically collect exposure, clinical status, covariates and multiple outcomes of interest; recruitment may include an unexposed cohort with the same indication. Pregnancy registries can be designed at the single product level, at the disease level, or as a multi-sponsor platform and may focus on products with or without a broad recommendation or indication for use during pregnancy. Benefits of a registry approach include near real-time data collection as soon as the product is commercially available and the ability to customize the data collection tool and potentially collect more detailed information. Limitations of this approach include challenges with patient recruitment and retention, difficulty in identifying an appropriate comparator, and potentially low statistical power to detect associations for rare outcomes.

**TABLE 2:** Overview of types of post-approval safety studies commonly conducted for vaccines in pregnant populations.

Study type	Type of data collection method	Study design	Common data source (non-exhaustive list)	Outcomes
Pregnancy Registry Studies	Primary	Prospective cohort	Questionnaires to pregnant people and HCPs	Maternal/pregnancy, fetal, neonatal
Electronic Data Source Studies	Secondary	Retrospective cohort Self-controlled case series	May include one or more: linked mother/baby healthcare insurance claims, EMR, disease registry, vital stats	Maternal/pregnancy, fetal, neonatal (note: may require validation with chart review/adjudication)
Passive Surveillance Studies	Primary	Generally mixed prospective and retrospective cohort	Spontaneous report data from pharmacovigilance database with additional questionnaires to HCPs of reported cases	Maternal/pregnancy, fetal, neonatal

Abbreviations: EMR, electronic medical records; HCP, healthcare provider.

Electronic data source studies can be designed as either a retrospective cohort study or a self-controlled case series and utilize secondary electronic healthcare data (e.g., insurance claims and/or electronic health records). These studies may be an efficient approach when valid pregnancy identification, exposure classification, mother-baby linkage, and outcome ascertainment can be established. Advantages of this approach include the ability to match exposed pregnancies to a control group of non-exposed, the availability of a denominator and possibility to conduct population-based assessments in large numbers of individuals. However, because these data sources were not designed for pregnancy research, these studies may encounter challenges in identifying pregnancies, capturing vaccine exposures, linking infants’ data for live births, estimating conception and gestational age, and ascertaining and validating study outcomes.

In scenarios when registries or database studies are not feasible or sufficient to generate evidence that can inform decisions (e.g., vaccines without a recommendation or indication for use in pregnancy with expected low uptake), passive surveillance studies can utilize exposed pregnancies identified through the routine pharmacovigilance (PV) mechanism with systematic, targeted follow-up by the MAH to assess pregnancy, fetal, and neonatal outcomes of interest; these single arm descriptive studies require contextualization from external sources. Given the voluntary nature of pregnancy exposure reports included in passive surveillance studies, the number of enrollees is difficult to predict. The final enrollment is generally low and depends on the frequency of vaccine exposure (e.g., recommended or not recommended) and reporting practices in the regions in which the vaccine is approved. There are limitations on comparators and denominators, and population-based analyses are not feasible. To enhance clarity and avoid confusion in this article, we differentiate between “pregnancy registry” for active prospective cohort studies and “passive surveillance study” for spontaneous reports, recognizing that some entities use different terms (e.g., some entities may refer to both study types as “registries”, or refer to passive surveillance studies as “enhanced passive surveillance studies”).

To further illustrate the scope of existing studies, a targeted review of examples of post-approval safety studies for vaccine exposures in pregnancy was conducted.

### 3.1 Methodology

The EU Post-authorisation Study (PAS) Register (now the “Heads of Medicines Agencies [HMA]-EMA Catalogue of Real-world Data [RWD] Studies”) was searched on 6 January 2023 to identify examples of post-approval safety studies in pregnancy after vaccine exposures. The following search criteria were applied:

- Study Type: Observational Study
- Population: Pregnant people
- Scope of Study: Risk Assessment

Among identified studies with a publicly available protocol, and/or report, an initial screening was done to select post-approval safety studies specific to vaccine safety in pregnant populations. A literature review was also conducted to supplement the identified studies; the search for studies was partly informed by the subject matter expertise of BeCOME members and was not a systematic literature review. This *ad hoc* approach was chosen to select 3-5 examples of studies representing each of the four following categories:

1. Studies of vaccines not broadly recommended for use in pregnancy using primary data collection
2. Studies of vaccines not broadly recommended for use in pregnancy using secondary data analysis
3. Studies of vaccines recommended for use in pregnancy using primary data collection
4. Studies of vaccines recommended for use in pregnancy using secondary data analysis

The following information was extracted from these studies from their publicly available protocols and/or publications, as available:

- Vaccine and indication
- Research question(s)
- Design and type of data collection (primary data collection vs. secondary data analysis)
- Data source
- Exposure window
- Study period
- Outcomes (including whether outcome validation was conducted)
- Sample size

### 3.2 Findings

Overall, 179 studies were initially identified from the EU PAS Register. Of those, only 19 studies had a publicly available protocol or study report available for review and were post-approval safety studies specific to the pregnant population. Among those, 14 studies evaluated vaccine exposures. To identify examples of studies representative of both “primary vs. secondary” and “vaccines recommended vs. not recommended for use in pregnancy”, six of the 14 studies were selected. Additionally, nine studies (including primary data collection studies, secondary database studies, and studies of vaccines both recommended or not recommended in pregnancy) were identified through supplementary literature searches, partly informed by the subject matter expertise of BeCOME members.

Of note, for this report, we defined vaccines “recommended for use in pregnancy” as those endorsed by at least one vaccine advisory committee (e.g., the ACIP) or deemed appropriate for use in pregnancy based on the benefit-risk assessment. We acknowledge that vaccine recommendations vary by country, meaning a vaccine recommended in one region may not be endorsed in others.

Study characteristics including product, study type, geographic scope, enrollment, and study durations are summarized in Tables 3–6. Studies were categorized by whether they assess vaccines recommended vs. not recommended for use in pregnancy and by primary data collection vs. secondary data analysis.

**TABLE 3:** Examples of post-approval safety studies in pregnancy for vaccines not broadly recommended for use in pregnancy (Primary data collection).

Vaccine	Indication summary	Objectives	Study Type/design	Study period^a^	Countries	Exposure period	Number enrolled	Number lost to Follow-up
Bexsero-MenB (GlaxoSmithKline, 2020a)	Prevention of invasive meningococcal disease caused by *Neisseria meningitidis* serogroup B for individuals 2 months of age and older (Europe) and for individuals 10 through 25 years of age (US)	Evaluate pregnancy outcomes among women immunized with the Bexsero vaccine within 30 days prior to the LMP or at any time during pregnancy	Registry (only exposed)	Jan 2016 - January 2019 (3 years)	US	From 30 days before LMP through end of pregnancy	2	2 (100%)
Cervarix-HPV (López-Fauqued et al., 2017; Angelo et al., 2014)	Prevention of premalignant ano-genital lesions and cervical and anal cancers causally related to certain oncogenic HPV types; use from the age of 9 years	Evaluate the risks of adverse pregnancy outcomes, including major teratogenic effects, in the offspring of women inadvertently exposed	Passive surveillance (only exposed)	Sep 2007 - November 2015 (∼8 years)	United Kingdom, US	From 60 days before conception date through end of pregnancy	306 reports (187 prospective and 45 retrospective reports)	74 (24%)
Gardasil-HPV/Silgard-HPV) (Goss et al., 2015)	Prevention of diseases caused by HPV types included in the vaccine; use from 9 through 26 years of age	Collect data regarding the safety profile of qHPV vaccine in pregnancy (inadvertent or off-label use)	Passive surveillance (only exposed)	Jun 2006 - December 2012 (∼7 years)	US, Canada, France	From 30 days before first day of the LMP through end of pregnancy	2,942 reports (2,566 prospective and 376 retrospective reports)	814 (32%) prospective reports and 14 (4%) retrospective reports
Varivax-VZV (Willis et al., 2022; Wilson et al., 2008)	Prevention of varicella in individuals 12 months of age and older	Monitor the pregnancy outcomes of women who inadvertently received varicella vaccine within 3 months before conception or at any time during pregnancy	Passive surveillance (only exposed)	Mar 1995 - October 2013 (∼19 years)	US, Canada	From 90 days before pregnancy through end of pregnancy	1,601 reports (1,522 prospective and 79 retrospective reports)	556 (37%) prospective reports and 2 (3%) retrospective reports

Abbreviations: Bexsero-MenB, Bexsero®-Neisseria meningitidis group B; Cervarix-HPV, Cervarix®-Human papillomavirus types 16 and 18; HPV, human papillomavirus; LMP, last menstrual period; Gardasil-HPV, Gardasil®-Human papillomavirus types 6, 11, 16, and 18; qHPV, quadrivalent human papillomavirus; US, united states; Varivax-VZV, Varivax®-varicella-zoster virus.

^a^ Study period refers to period of enrollment and/or data collection. Follow-up length varied considerably across studies and was inconsistently reported. When follow-up was mentioned, it ranged from none to 24 months post-delivery.

**TABLE 4:** Examples of post-approval safety studies in pregnancy for vaccines not broadly recommended for use in pregnancy (Secondary data analysis).

Vaccine	Indication summary	Objectives	Study Type/design	Study period^a^	Countries/Data Source	Exposure period	Study population size
Trumenba -Men B (Pfizer Inc, 2021)	Prevention of invasive disease caused by *Neisseria meningitidis* serogroup B in individuals 10 through 25 years of age	Estimate incidence and risk ratios of pregnancy outcomes in women exposed and not exposed to Trumenba in up to 28 days prior to or during pregnancy; and estimate prevalence and risk ratios of birth outcomes (major congenital anomalies) among infants exposed and not exposed to Trumenba *in utero* (planned objectives)	Cohort (Initially including an unexposed cohort, but converted into a descriptive study given the small number of Trumenba exposures identified in the first phase of the study)	Nov 2015 - December 2020 (∼5 years)	US Electronic healthcare data and linked birth certificates from four Sentinel Research Partners^b^	From 28 days before LMP through end of pregnancy (for live births/stillbirths) or through 20 weeks (for spontaneous abortions)	51 exposed pregnancies (initially projected to range between 468 and 936)
Menveo-MenACWY (Becerra-Culqui et al., 2020)	Prevention of invasive meningococcal disease caused by *Neisseria meningitidis* serogroups A, C, Y, and W-135 in individuals 2 months through 55 years of age	Describe pregnancy and birth outcomes among pregnant women inadvertently vaccinated with MenACWY-CRM as part of routine care	Cohort (Exposed only) Estimates of exposed population were compared with prevalence reported in the CDC National Vital Statistics System and other literature	Sep 2011 - June 2013 (∼2 years)	US Kaiser Permanente Southern California	From 28 days before conception date through end of pregnancy	92
Cervarix-HPV (Baril et al., 2015; GlaxoSmithKline, 2013)	Prevention of premalignant ano-genital lesions and cervical and anal cancers causally related to certain oncogenic HPV types from the age of 9 years	Asses the risk of spontaneous abortion after inadvertent exposure to HPV-16/18-vaccine during pregnancy	Cohort (Exposed and unexposed)	Sep 2008 - June 2011 (∼3 years)	United Kingdom Clinical Practice Research Datalink General Practice Online Database	First day of gestation between 30 days before and 45 days after vaccination (or between 30 days before and 90 days after vaccination for an extended period)	207 in the exposed cohort (330 for the extended risk period) and 632 in the unexposed cohort

Abbreviations: CDC, centers for disease control and prevention; Cervarix-HPV, Cervarix®-Human papillomavirus types 16 and 18; HPV, human papillomavirus; Trumenba-MenB, Trumenba®-Neisseria meningitidis group B; LMP, last menstrual period; Menveo-MenACWY, Menveo®-Neisseria meningitidis groups A, C, W, and Y; US, united states.

^a^ Study period refers to period of enrollment and/or data collection. Follow-up length varied considerably across studies and was inconsistently reported. When follow-up was mentioned, it ranged from none to 24 months post-delivery.

^b^ Harvard Pilgrim Healthcare Institute, CVS, Health Clinical Trial Services (Aetna), HealthCore, Inc. (Anthem), and Meyers Primary Care Institute.

**TABLE 5:** Examples of post-approval safety studies for vaccines recommended for use in pregnancy (Primary data collection).

Vaccine	Indication summary	Objectives	Design	Study period^a^	Countries	Exposure period	Number enrolled	Number lost to Follow-up
Fluzone IIV4-Influenza^b^ (Ledlie et al., 2022)	Prevention of influenza A and B	Monitor exposure during pregnancy and any corresponding maternal, pregnancy, obstetrical and neonatal outcomes using routine pharmacovigilance surveillance	Passive surveillance (only exposed)	Aug 2013 – September 2019 (∼6 years)	9 countries (85% from US, Australia, Canada)	From 30 days before LMP through end of pregnancy	239 (210 prospective; 29 retrospective)	148 (62%)
Fluarix IIV4/FluLaval IIV4-Influenza (GlaxoSmithKline, 2020b)	Prevention of influenza A and B	Describe the characteristics of prospectively reported pregnancies with abnormal pregnancy outcomes Assess the proportion of prospectively reported pregnancies with abnormal pregnancy outcomes	Passive surveillance (only exposed)	Jun 2014 - May 2019 (∼5 years)	US	From 28 days before conception date through end of pregnancy	507 (40 ongoing)	352 (69%)
Adacel-Tdap^c^ (Switzer et al., 2021)	Prevention of tetanus, diphtheria, and pertussis	Capture information on pregnancy exposures, as well as on maternal, obstetrical, pregnancy and neonatal outcomes to monitor for any potential safety signals	Passive surveillance (only exposed)	Jun 2005-October 2016 (∼11 years)	Mostly US, with a few reports from 10 other countries	During pregnancy	1,181 (1,167 prospective; 15 retrospective)	837 (70.8%)
Comirnaty-COVID-19 (Pfizer Inc, 2022)	To prevent COVID-19 caused by SARS-CoV-2	Assess whether pregnant women who received the vaccine during pregnancy experienced increased risk of pregnancy and infant safety outcomes relative to pregnant women who received no COVID-19 vaccines during pregnancy	Registry (Exposed and unexposed)	Dec 2020 - June 2024 (∼4 years)	US, Canada	From 1 month before first day of the LMP through end of pregnancy	Target 2,000 (1,100 exposed and 900 unexposed)	NA
Multiple manufacturers- COVID-19 vaccines (Wyszynski et al., 2023)	To reduce morbidity and mortality from COVID-19	To estimate the risk of obstetric, neonatal, and infant outcomes among pregnant people and their offspring who were exposed to a COVID-19 vaccine during pregnancy relative to a country- and gestational-age-matched reference group of people who received no COVID-19 vaccines during pregnancy	Registry (Exposed and unexposed)	2021–2026 (5 years)	24 countries	From 30 days before first day of the LMP through end of pregnancy	Target 6,000 (at least 500 per vaccine brand)	NA

Abbreviations: Adacel-Tdap, Adacel®-Tetanus, diphtheria, and acellular pertussis vaccine; Comirnaty-COVID-19, Comirnaty®- Coronavirus disease 2019 mRNA, vaccine; COVID-19, coronavirus disease 2019; Fluarix IIV4/FluLaval IIV4-Influenza, Fluarix^®^, FluLaval^®^, Inactivated Influenza Vaccines, Quadrivalent; Fluzone IIV4-Influenza, Fluzone^®^, Inactivated Influenza Vaccines, Quadrivalent; LMP, last menstrual period; Multiple manufacturers-COVID-19, vaccines, Coronavirus disease 2019 vaccines; NA, not available; SARS-CoV-2: severe acute respiratory syndrome coronavirus 2; US, united states.

^a^ Study period refers to period of enrollment and/or data collection. Follow-up length varied considerably across studies and was inconsistently reported. When follow-up was mentioned, it ranged from none to 24 months post-delivery.

^b^ The post-approval commitment study have been completed (Study Details | Sanofi Pasteur Quadrivalent Influenza Vaccine (QIV) Pregnancy Registry | ClinicalTrials.gov); however, the passive surveillance program remains open and accessible on the company website (https://www.sanofivaccinespregnancyregistry.com).

^c^ Representing results from an interim data cut, this study is still ongoing.

**TABLE 6:** Examples of post-approval safety studies for vaccines recommended for use in pregnancy (Secondary data analysis).

Vaccine	Indication summary	Objectives	Study Type/design	Study period^a^	Countries/Data source	Exposure period^a^	Study population size
Spikevax-COVID-19 (Moderna, 2021)	To reduce morbidity and mortality from COVID-19	Determine whether exposure to the Spikevax during pregnancy is associated with an increased risk of pregnancy complications, adverse pregnancy outcomes, major congenital malformations in the offspring (overall and organ-specific if feasible), and adverse neonatal outcomes	Retrospective cohort (exposed and unexposed)	Jan 2021 - December 2022 (∼2 years)	Denmark, Italy, Norway, Spain, United Kingdom EMR, national registry, administrative claims	During pregnancy	No specific target. However, estimated 20,000 to 100,000 live births, depending on data source
Abrysvo-RSV (Pfizer and Inc, 2024)	Prevention of RSV-associated lower respiratory tract illness in infants from birth up to 6 months of age by active immunization of pregnant individuals	1. Estimate the risk of 1) preterm birth and 2) pregnancy-associated hypertensive disorders following exposure to ABRYSVO during pregnancy, overall and among pregnant individuals who are immunocompromised 2. Estimate the risk of additional safety outcomes of specific interest following exposure to Abrysvo during pregnancy, overall and among pregnant individuals who are immunocompromised	1. Rapid cycle analysis (historical and concurrent comparator groups exposed to influenza, Tdap, or COVID-19 vaccinations) 2. Retrospective cohort (exposed and unexposed)	Jan 2023 - September 2024 (∼2 years) for retrospective cohort	US Research partners who also contribute to the US FDA’s Sentinel Initiative	During weeks 32 through 36 of gestation (approved gestational ages)	All pregnancies among individuals who meet the study inclusion/exclusion criteria during study period eligible
Boostrix-Tdap (Tseng et al., 2022; Florea et al., 2023)	Prevention of tetanus, diphtheria, and pertussis	Evaluate the safety of prenatal tetanus, diphtheria, acellular pertussis (Tdap) vaccination administered on or after the first day of the 27th week of pregnancy	Retrospective matched cohort (exposed and unexposed)	Jan 2018 - January 2019 (exposed cohort; 1 year); January 2012 - December 2013 (unexposed cohort; ∼2 years)	US Kaiser Permanente Southern California	From week 27 of gestation through delivery (recommended gestational ages by ACIP)	33,212 (16,606 pairs of Tdap recipients and unexposed pregnant women)

Abbreviations: Abrysvo-RSV, Abrysvo®-Respiratory Syncytial Virus vaccine; Boostrix®-Tetanus, diphtheria, and acellular pertussis vaccine (Boostrix-Tdap); CIP, advisory committee on immunization practices; EMR, electronic medical record; FDA, food and drug administration; RSV, respiratory syncytial virus; Spikevax®- Coronavirus disease 2019 mRNA, vaccine (Spikevax-COVID-19); Tdap = tetanus, diphtheria, acellular pertussis; US, united states.

^a^ Study period refers to period of enrollment and/or data collection. Follow-up length varied considerably across studies and was inconsistently reported. When follow-up was mentioned, it ranged from none to 24 months post-delivery.

## 4 Discussion

### 4.1 Study type

#### 4.1.1 Overview of existing studies

The reviewed studies (Tables 3–6) varied widely in their design approaches and included examples of the three overarching study types described in Table 2: pregnancy registry studies, electronic data sources (i.e., secondary data analysis), and passive surveillance studies. In this review, secondary data analysis studies were used for both vaccines recommended for use in pregnancy and not broadly recommended for use in pregnancy. Passive surveillance studies were particularly common for vaccines that were not broadly recommended to be administered during pregnancy. Among the studies of vaccines not broadly recommended for use in pregnancy, the passive surveillance studies tended to have larger sample sizes (range: 306–2,942) relative to the other designs (range: 2–207). Additionally, as anticipated, actual and target sample sizes (reported for newer studies without publicly available results at the time of the search) tended to be higher among studies for vaccines recommended to be administered during pregnancy, as compared to those not broadly recommended to be administered during pregnancy.

#### 4.1.2 Current guidance

The FDA guidance refers to registries being based on the voluntary participation of individuals who have been exposed to a specific drug or biological product (including vaccines) during pregnancy and unexposed individuals who enroll into the comparator cohort. For products that are anticipated to be used rarely during pregnancy (e.g., treatment of advanced cancer), the FDA guidance mentions that the sponsor “can consider a pregnancy surveillance program (i.e., a structured approach for data collection with targeted questionnaires to obtain follow-up information on all exposed pregnancies of which sponsors become aware).” These pregnancy surveillance programs usually need worldwide safety data collection “to identify a sufficient number of exposed pregnancies for clinical safety assessment.” This guidance document also stresses that while pregnancy registries are important, their design usually makes them insufficient for use in isolation, and therefore, the FDA recommends a complementary approach of using a registry and another method (e.g., an electronic database study).

The EMA guidance documents note that existing databases and/or existing disease-specific registries are recommended whenever possible, however do not recommend a specific study design (including for non-vaccine studies) nor offer vaccine-specific recommendations. Features of different study designs are described and references to the GVP Module VIII (European Medicines Agency, 2017) and CHMP Guideline on the Exposure to Medicinal Products During Pregnancy: Need for Post-authorisation Data (European Medicines Agency, 2005) are made.

#### 4.1.3 Actionable Recommendations

The current FDA guidance section “Pregnancy Registries/Overview” does not include vaccine-specific recommendations which address the challenges unique to vaccine studies. Additional guidance on the criteria for determining the most appropriate design for vaccine studies is desirable. For vaccines recommended during pregnancy, in which the number of exposures are generally expected to be large, establishing a registry with active enrolment seems appropriate. For vaccines not recommended during pregnancy, mainly inadvertent exposures (particularly before pregnancy or during the first pregnancy trimester) are expected. For these vaccines, the choice of the most appropriate design is less straightforward. In some cases, the number of exposed pregnant individuals may not be negligible if the vaccine is widely administered in a population of childbearing potential. For other vaccines, the number of exposed pregnant individuals may be expected to be low, and thus setting up a registry with active recruitment may face feasibility challenges.

Additionally, the current guidance lacks clarity on specific factors that determine when pregnancy registries conducted via passive surveillance (e.g., passive surveillance studies through spontaneous case reporting in PV database) are appropriate as compared to active pregnancy registries with active patient recruitment and data collection. There is a need for guidance to define criteria for selecting passive surveillance studies versus active pregnancy registries based on rarity of exposure, availability of clinical trial data, vaccine type (e.g., yearly influenza strain updates), vaccine coverage, existing data robustness, pregnancy recommendations, contraindications, and the feasibility of active surveillance implementation.

### 4.2 Comparators

#### 4.2.1 Overview of example studies

Of the reviewed example studies (Tables 3–6), those designed as passive surveillance pregnancy studies based on spontaneous reporting all lacked comparison groups (for vaccines with or without recommendation for use in pregnancy). In contrast, most of the reviewed cohort studies with active data collection, whether prospective or retrospective, included a comparison group. The comparators consisted of individuals who either received other vaccines during pregnancy or were exposed to the vaccine under study before becoming pregnant. In the studies that did not include internal comparison groups, the estimates of the exposed pregnant people were compared to background population estimates.

Studies using secondary data analysis for vaccines not widely recommended during pregnancy seemed to face more challenges in identifying internal comparator groups. For instance, one study on the safety of Boostrix-Tdap (Tseng et al., 2022) selected a historical unexposed control cohort rather than a concurrent one, as the vaccine uptake among pregnant people exceeded 80%, which impaired the feasibility of 1:1 matching and could introduce confounding by indication.

#### 4.2.2 Current guidance

Both the FDA and EMA guidelines encourage the use of multiple comparator groups in pregnancy studies. Specifically, the FDA guidance recommends the use of a concurrent internal comparison group of pregnant people who are unexposed to the treatment under study. While the FDA guidance acknowledges that in certain situations, background rates from another pregnancy registry or external database may be the only available comparator, it cautions that this approach has limitations that should be considered when interpreting results.

#### 4.2.3 Actionable recommendations

Both the FDA and EMA guidelines provide general recommendations for comparators in pregnancy safety studies and lack specific guidance for vaccine pregnancy studies, particularly for studies utilizing secondary data analysis. While these guidelines highlight the advantages of disease-specific pregnancy registries over product-specific ones, as the former include built-in comparator groups and can mitigate the risk of confounding by indication, these registries have limited utility in preventive vaccine studies. The FDA guidance emphasizes the importance of selecting appropriate comparators—warning that “comparing dissimilar populations could bias the study results, indicate a risk when none exists, or mask an increased risk that exists”—however, detailed recommendations relevant for vaccine studies are notably absent.

The comparator group could consist of unexposed pregnant people, although this approach must carefully address potential unmeasured confounding as the decision to receive or decline vaccination may correlate with health behaviors, socioeconomic factors, or underlying health conditions that affect pregnancy outcomes. Background rates in the target population can serve as comparators. These rates can be either concurrent with vaccine exposures or from periods before vaccination campaigns began, though each approach has limitations. Concurrent rates may be “contaminated” by vaccine effects if a large proportion of the population is vaccinated, while historical rates might be influenced by temporal changes in diagnostic practices and reporting systems. Furthermore, the availability and quality of pre-vaccination historical data often presents a significant challenge, potentially limiting the reliability of historical comparisons. Alternatively, comparisons may include pregnant people who received another vaccine during pregnancy, though this approach is increasingly challenging due to the growing number of recommended multi-component vaccines during pregnancy. More comprehensive recommendations on the selection of preferred and appropriate comparators for vaccine safety studies would be beneficial to stakeholders.

### 4.3 Exposure period

#### 4.3.1 Overview of example studies

We observed varying approaches to defining exposure periods (Tables 3–6). Ten studies, regardless of data collection methodology or pregnancy use indication, considered vaccine exposure during a brief period (typically 1 month) before pregnancy start and throughout the entire pregnancy. For studies that differed from this approach, vaccine exposure was defined in several ways: administration of a vaccine dose at any time during pregnancy (Adacel-TdaP, Spikevax-Covid-19 studies); administration of a vaccine dose within a defined period during pregnancy, either related to approved gestational ages (Abrysvo-RSV) or gestational ages as recommended by the Advisory Committee on Immunization Practices (ACIP) in the US (Cervarix-HPV/secondary data; Table 4). In general, the studies defined pregnancy start at the last menstrual period (LMP) date or conception date.

Only three studies explicitly described their approach to handling multiple vaccine doses (Cervarix-HPV study/primary data, Trumenba-MenB, Varivax-Varicella). The Cervarix-HPV study (primary data; Table 3) classified multiple exposures during pregnancy by the earliest trimester of exposure, and when exposure occurred both before and after conception, classification was based on the post-conception dose. In two other studies (Trumenba-MenB, Varivax-Varicella), multiple doses were possible during the exposure period, with pregnant individuals considered exposed at the time of the first vaccine dose. Of note, a multi-dose immunization schedule is not recommended for all vaccines, and for single dose regimens, multiple vaccine doses may indicate administration errors.

Eight studies considered strata of timing of exposure in the analysis, such as a month before LMP and/or trimesters of gestation (Varivax-Varicella, Cervarix-HPV/primary data Table 3, Trumemba-MenB, Menveo- MenACWY, Fluarix IIV4/FluLaval IIV4-Influenza, Comirnaty-Covid-19, Spikevax-Covid-19, Abrysvo-RSV).

#### 4.3.2 Current guidance

The EMA Guidance emphasizes the importance of accurate exposure measurement during embryo-fetal development (precise timing for both pregnancy start and medicine use) and gestational age at time of exposure. It describes susceptible periods for adverse pregnancy outcomes based on the gestational age at time of exposure. These periods include (1) the first 2 weeks after conception, when interference may result in early pregnancy loss; (2) gestational weeks 4–16, which are critical to study the association of exposure and major birth defects, though each congenital abnormality has its specific critical period (e.g., neural tube defect between the gestational days 29 and 42 or between days 15 and 28 post-conception); (3) week 16 until delivery, when interference may cause minor anomalies, growth impacts, or functional defects; (4) late pregnancy^3^ and delivery, which carry risks of physiological impacts on the neonate. Finally, throughout the entire pregnancy, exposure to environmental agents, including medicines, as well as exposure to infections or worsening of existing chronic diseases, can result in pregnancy loss or stillbirth.

The FDA Guidance advises collecting detailed information on medication use (including start/stop dates, dose, frequency, duration, and indication), incorporating exposure information from the period just before pregnancy (especially for products with long half-lives), and conducting stratified analyses by gestational timing of exposure, with a separate analysis of first-trimester exposures for MCMs. For complementary (e.g., secondary database) studies, the FDA Guidance emphasizes accurate gestational age estimation, suggesting various methods for identification (e.g., birth certificates, ICD codes, electronic health records, ultrasound), and recommends obtaining informed consent for medical record review in population-based case-control studies to confirm diagnoses and collect additional exposure data.

Both EMA and FDA guidance recommends recording exposure to any other medical product during pregnancy as well as the medical history and other potential confounders.

#### 4.3.3 Actionable recommendations

A notable gap exists in both FDA and EMA guideline documents, which lack recommendations for designing pregnancy studies specific to vaccines. Current recommendations primarily focus on drug use parameters such as start/stop dates, frequency, and duration, which are not fully applicable to characterize vaccine exposure. Critical aspects of vaccine exposure documentation include brand name, batch number, and dose number when it is part of an immunization series against the same disease (DeSilva et al., 2016). This set of data is essential for comprehensive exposure assessment but remains unaddressed in current regulatory guidance. Additional guidance on the critical elements to characterize vaccine exposure is required.

It is widely recognized that the first trimester is the most critical period for exposure during pregnancy with regards to subsequent effects on fetal development (teratogenicity). Both FDA and EMA guidelines emphasize the importance of considering specific timing of exposure when evaluating outcomes of MCMs. The EMA guidance provides insights on the relationship between exposure timing and gestational age regarding the risk of MCMs and other adverse outcomes, detailing susceptible periods throughout pregnancy. However, DeSilva et al. (2016) offer more precise recommendations that could enhance regulatory guidance; these authors suggest including maternal vaccination from 30 days prior to conception up to 20 weeks gestational age. The rationale for this time frame is to account for wider windows of teratogen exposure and potential errors in assigning conception dates and gestational age.

It is worth noting that different exposure windows may be relevant for certain outcomes and should be considered accordingly. Additional regulatory guidance on defining appropriate exposure periods for vaccines would be valuable for researchers conducting vaccine safety studies during pregnancy.

### 4.4 Outcomes

#### 4.4.1 Overview of example studies

Across all 15 studies identified, we describe in Table 7 the alignment of the prespecified outcomes in the study protocol with the EMA (European Medicines Agency, 2019; The European Network of Centres for Pharmacoepidemiology and Pharmacovigilance, 2010; European Medicines Agency, 2005), FDA (United States Food and Drug Administration, 2019), and Brighton Collaboration guidelines (Munoz et al., 2015; Jones C. E. et al., 2016) for both primary and secondary data designs. Many of the primary data collection studies were passive surveillance studies, making it challenging to identify target outcomes due to the lack of an *a priori* list. In these cases, only outcomes specifically described as outcomes of interest were summarized.

**TABLE 7:** Evaluation of post-approval safety studies in pregnancy: alignment of outcomes with EMA, FDA, and brighton collaboration guidelines in primary and secondary data.

Outcome	Outcome explicitly recommended by the EMA (European Medicines Agency, 2019; The European Network of Centres for Pharmacoepidemiology and Pharmacovigilance, 2010; European Medicines Agency, 2005)	Outcome explicitly recommended by the US FDA (United States Food and Drug Administration, 2019)	Brighton collaboration priority outcomes^a^ (Munoz et al., 2015)	N (%) of Example studies collecting the outcome explicitly	
				Primary data collection studies (N = 9)	Secondary data analysis studies (N = 6)
Pregnancy or maternal outcomes					
Spontaneous abortion/Miscarriage	Yes	Yes	No^b^	9 (100%)	4 (67%)
Elective Termination/Induced abortion for any reason	Yes	Yes	No^b^	7 (78%)	3 (50%)
Termination of Pregnancy due to Fetal Anomaly	Yes	Yes	No	0 (0%)	1 (17%)
Ectopic pregnancy	Yes	No	No	3 (33%)	1 (17%)
Molar pregnancy	Yes	No	No	1 (11%)	0 (0%)
Maternal death	Yes	No	Yes	0 (0%)	2 (33%)
Fetal and neonatal outcomes					
Live Birth	Yes	Yes	No^b^	7 (78%)	3 (50%)
Fetal Death/Stillbirth	Yes	Yes	Yes	9 (100%)	5 (83%)
Congenital Malformation/Anomalies	Yes	Yes	Yes	9 (100%)	5 (83%)
Small for Gestational Age	Yes	Yes	Yes	2 (22%)	5 (83%)
Low birth weight	Yes	Yes	Yes	5 (56%)	3 (50%)
Preterm delivery/birth	Yes	Yes	Yes	6 (67%)	6 (100%)
Neonatal dysmaturity	Yes	No	No	0 (0%)	0 (0%)
Neonatal illness	Yes	No	No	1 (11%)	0 (0%)
APGAR score	Yes	Yes	No^b^	2 (22%)	1 (17%)
Fetal growth restriction	Yes	No	Yes	1 (11%)	1 (17%)
Perinatal, neonatal, infant death	No	No	Yes	1 (11%)	4 (67%)
Child outcomes					
Developmental milestones	Yes	Yes	No^b^	2 (22%)	0 (0%)

Abbreviations: FDA, food and drug administration; EMA, european medicines agency; US, united states.

^a^ According to the Brighton collaboration global consultation, “priority outcomes” represent the most critical terms selected for evaluating vaccine safety during pregnancy. This is a non-exhaustive list; other “priority outcomes” not explicitly recommended by EMA or FDA, as well as “outcomes” considered important (but not critical) and “enabling” terms (used to assist in the assessment of other outcomes or priority outcomes) are not listed here.

^b^ While not a priority outcome, this outcome is defined by Brighton collaboration. For example, Live birth is part of neonatal key term and defined as enabling term or outcome; Development milestones are defined as suggested outcomes for neurodevelopmental disability.

Almost all studies included outcomes for fetal death/stillbirth (N = 14), major congenital malformations (MCMs; N = 14), and spontaneous abortion/miscarriage (N = 13). Fewer, but still a majority, included an outcome for preterm delivery (N = 12), elective termination/induced abortion for any reason (N = 10), low birth weight (N = 8), and small for gestational age (N = 7). Few studies evaluated maternal death (N = 2), ectopic pregnancies (N = 4), and molar pregnancies (N = 1). Developmental milestones were captured in two studies, but only up to a maximum of 1 year. Other endpoints not specifically recommended by health authorities (e.g., preeclampsia, premature rupture of membranes, gestational diabetes, postpartum hemorrhage, gestational hypertension, cesarean delivery, placenta previa, and large for gestational age) were collected inconsistently across studies. Additional adverse events (AEs) and serious adverse events (SAEs) may have been collected and reported in accordance with routine PV practices, in addition to the prespecified outcomes, as established within the primary data collection studies.

Overall, there was wide variability in identified outcomes. All nine primary data collection studies collected information on congenital anomalies; however, only three reported that these anomalies were reviewed by an independent expert, with two utilizing a dysmorphologist (Cervarix-HPV; Multiple manufacturers-COVID-19 vaccines). Only one study (Comirnaty-COVID-19) explicitly adhered to the FDA draft guidance, where an expert dysmorphologist, serving as a co-investigator for the OTIS Pregnancy Registry studies, reviewed all medical records and reports of MCMs. This review process was blinded to exposure status and consistently applied across both exposed and comparator cohorts, aligning with FDA recommendations.

Among the six secondary data analysis studies, five studies assessed congenital malformations and, of those, three documented varying validation approaches. Two studies implemented expert review: the Menveo-MenACWY study utilized a birth defect evaluator with genetics expertise serving on an independent Scientific Advisory Committee and applying the Centers for Disease Control and Prevention (CDC’s) Metropolitan Atlanta Congenital Defects Program (MACDP) definitions, while the Cervarix-HPV study employed adjudication by two independent external teratology experts who remained blinded to exposure status. The Boostrix-Tdap study relied on chart confirmation of AEs. These approaches reflect varying adherence to FDA guidance, which recommends outcome validation through clinical expert review, linkage to birth defect registries, or previously validated high-performing algorithms in the same (or similar) database planned for the current analysis.

#### 4.4.2 Current guidance

The Brighton Collaboration (Munoz et al., 2015) has defined a consensus list of terms and concept definitions of key events for monitoring immunization in pregnancy through a rigorous process involving systematic literature review, worldwide stakeholder survey, and taskforce meetings. Priority outcomes were defined as those that are key for safety assessment of vaccine studies in pregnancy (summarized in Table 7). No priority outcomes were provided for childhood outcomes, as maternal vaccination effects on older children were deemed unlikely. Of note, guidance for assessing neurodevelopmental delay was later added to the Brighton Global Alignment of Immunization Safety Assessment in pregnancy [GAIA] Case Definitions (Villagomez et al., 2019).

The FDA guidance (summarized in Table 1) does not provide a specific list of outcomes but includes recommended data collection elements in Appendix A under different subsections (General, Maternal information, Neonatal information) which can be used to deduce relevant outcomes. While the FDA guidance is mostly tailored to a primary data collection study design, it is noted that the list of outcomes may also be applicable to complementary studies (e.g., studies with a secondary collection design). Furthermore, the FDA provides guidance on outcome ascertainment and validation in study designs relying on secondary data (including electronic administrative claims data and/or electronic health record databases), recommending validation using a gold standard method such as via medical record review from clinical experts or via linkage to birth defect registries and/or birth certificate data, or through a high-performing algorithm validated for the specific outcome in the same or similar database.

EMA guidance documents (summarized in Table 1), indicate that all “relevant outcomes throughout the human developmental cycle […] with child follow up for a long enough period to capture relevant information […]” should be evaluated and offer a summary of commonly studied outcomes. The EMA guidance also suggests monitoring longer-term child outcomes such as neurodevelopmental disorders but provides no guidance for outcome validation.

Other initiatives, such as the effort coordinated by St George’s, University of London, have leveraged the comprehensive work of harmonized case definitions for key obstetric and neonatal outcomes, done by the GAIA project and the Brighton Collaboration network to provide comprehensive guidance in harmonizing the data collected in case report forms (CRFs) for clinical trials (Jones C. E. et al., 2016). However, there are still gaps. The CRF data collection guidance is best suited for primary data collection in clinical trials. While it can be adapted for use in post-approval secondary data studies, the limitations of secondary use of data preclude full implementation. Furthermore, while several of the harmonized priority data collection elements recommended in the guidance can be mapped to the EMA and FDA guidance outcomes, there are a few EMA/FDA recommended outcomes that cannot be directly mapped to the CRF data elements (Jones C. et al., 2016). For example, the data collection guidance does not specifically indicate that elective termination/induced abortion for any reason, and termination of pregnancy due to fetal anomaly (both of which are recommended for collection in the EMA and FDA guidance documents) should be collected. Although it recommends that information on “any new medical conditions, surgical procedures or hospitalizations” during pregnancy be collected. These open-ended questions lead to challenges in harmonization.

#### 4.4.3 Actionable Recommendations

While both regulatory agencies emphasize that studies should capture both live and non-live birth pregnancy outcomes, and recommended or commonly studied outcomes are listed, neither regulatory guidance document outline a specific subset of outcomes that all studies must evaluate. Additional guidance that provides (1) a comprehensive list of a subset of outcomes that all studies must evaluate across indications and, within this list of required outcomes, (2) a prioritization of which outcomes are most important and thus should drive calculations of sample size and study design, would be greatly beneficial to ensure consistency across similar studies, allow for meaningful comparisons, and clearly communicate expectations. For studies requiring additional outcomes beyond the prioritized list, a decision framework for selecting the appropriate outcomes is needed.

Additional guidance is needed regarding when and how long follow-up is needed, and the appropriate strategies for assessing childhood outcomes (e.g., growth and development). As this is likely to be dependent on factors such as the vaccine, disease, and setting, a decision framework would be useful in determining relevance for a given study.

Validation is crucial for ensuring data reliability but should be undertaken judiciously, guided by clear criteria delineating when it is necessary. In cases where existing validated algorithms have been successfully employed in analogous or identical data sources, it is prudent to utilize these established methodologies. This approach obviates the need for redundant efforts in developing new algorithms, advocating instead for the consistent application of proven processes. Such consistency would enhance the strength and reliability of data and conclusions. Nonetheless, it is acknowledged that these algorithms may require updates and adaptations to specific studies. While the FDA has outlined expectations regarding validation of study outcomes, achieving harmonization in validation expectations across regulatory authorities remains an essential goal.

Electronic databases are increasingly utilized for pregnancy safety studies. In these data sources, researchers often rely on algorithms to identify cases. The integration of innovative machine learning techniques for improving algorithms may offer opportunities to refine case definitions. Continued methodological research in this area may further strengthen the validity and reliability of case definitions. Guidance on the use of machine learning techniques to refine case definitions would be of value.

### 4.5 Enrolment target/sample size

#### 4.5.1 Overview of example studies

An important practical hurdle in pregnancy research is achieving adequate sample sizes as vaccine exposure may be uncommon, and some maternal, fetal or neonatal outcomes of interest are rare. Sample sizes in identified studies (Tables 3–6) varied widely: from 2 to 2,942 exposures for vaccines not recommended in pregnancy, and from 239 to about 16,606 exposures for vaccines recommended for use in pregnancy.

#### 4.5.2 Current guidance

The FDA guidance mentions that for pregnancy registries, the “determination of an adequate sample size depends on the objective(s) and design of the registry and the background rate of the outcome in the study population. If more than one pregnancy outcome is considered, sample size determination should be based on the outcome with the lowest background rate (e.g., MCM).” It also notes that a single teratogen is unlikely to increase the risk of all MCMs and acknowledges that pregnancy registries often lack power to study specific MCM risks unless the effect size is substantial.

The EMA guidance suggests pooling data from multiple databases to address sample size concerns. The sample size should be adequate to evaluate single events of birth defects rather than all MCMs aggregated.

The sample size calculation is also impacted by the exposure period. Indeed, as the window of exposure gets smaller, fewer pregnancies meet the exposure criteria, thus reducing the sample size in the exposed cohort. The FDA guidance mentions that “typically, a specific defect or pattern of defects is associated with a specific teratogenic exposure during a critical period”, which generally occurs during the first trimester. In earlier FDA guidance (United States Food and Drug Administration, 2005) this is further described, stating that “each part, tissue, and organ of an embryo has a critical period during which its development may be disrupted” and that “evaluating the timing of exposure is also important when assessing the power of a study.”

#### 4.5.3 Actionable recommendations

Frequent adverse outcomes include preterm birth (10% of US infants in 2022) (CDC, 2025a), and small for gestational age (11.1% of US live births) (Jensen et al., 2019). Major structural or genetic birth defects affect 3% of births in the US (Centers for Disease Control and Prevention, 2008); prevalence estimates ranged from 0.63 per 10,000 live births for common truncus to 18.65 for clubfoot (Stallings et al., 2024) during 2016–2020. Other outcomes of interest are rare (e.g., stillbirth, 5.7 per 1,000 births (CDC, 2025b)). For each outcome, the minimal sample size to be reached can be determined, based on the prevalence in the comparison group, the desired minimal detectable risk, and the statistical power. A pregnancy safety study assessing multiple outcomes of interest will have a variable minimal detectable risk per outcome for constant power and sample size.

Additional guidance on minimal detectable risk and statistical power for priority outcomes is essential to ensure that studies are being designed to meet the targets expected by regulators. The requirements may differ based on the state of knowledge (no specific risk identified versus specific safety concern), which will inform the selection of the study objectives, and based on the expected level and timing of exposure, impacting study feasibility.

### 4.6 Study duration and criteria to close a post-approval registry

#### 4.6.1 Overview of example studies

We observed varying durations for data collection across studies (Tables 3–6), with primary data collection studies spanning three to 19 years and secondary data studies spanning one to 5 years. These differences were driven by sample size targets and differences in vaccine exposure among pregnant persons (e.g., indications, recommendations, and uptake rates in participants of childbearing potential and pregnant persons). Many studies, particularly passive surveillance studies, did not have pre-specified enrolment targets. These studies are typically established as post-approval requirements or commitments with regulatory agencies, requiring periodic reports (e.g., annually for 5 years) and a final report upon study completion. In some cases (e.g., FDA Fluarix (US Food and Drug Administration, 2012)), regulators may require continued enrollment until they approve study closure, leading to extended durations. In the case of varicella zoster virus (VZV)-containing vaccines^4^, two factors limited the study’s feasability: the low exposure rate among varicella-susceptible women of childbearing age and the rarity of congenital varicella syndrome. These made it unlikely to collect robust data within a reasonable timeframe (Marin et al., 2014). As a result, the FDA supported closing the registry and revising product label information. The registry, initiated in March 1995, ceased patient enrollment in October 2013.

#### 4.6.2 Current guidance

Regulatory guidelines do not address the duration of the inclusion period, which is tied to target sample size, the feasibility of achieving it within reasonable timelines, and participant retention. The FDA recommends pregnancy registries continue until one or more of the following occurs: “(1) sufficient information has accumulated to meet the scientific objectives of the registry, (2) the feasibility of collecting sufficient information diminishes to unacceptable levels due to low exposure rates, poor enrollment, or loss to follow-up, and (3) other methods of gathering appropriate information become achievable or are deemed preferable.”

The study duration is also determined by the outcomes of interest. Longer-term outcomes, such as child health, growth, and neurodevelopment, require extended follow-up duration. Some cardiac, renal, and intestinal malformations may not be diagnosed immediately postpartum, and their incidence is significantly influenced by the duration of follow-up and diagnostic tests availability. Therefore, long-term follow-up is recommended when possible and appropriate. According to the EMA GVP guidelines, the decision to pursue long-term follow-up for childhood outcomes should be based on biological plausibility and supporting evidence from available data such as non-clinical findings, clinical outcomes, pharmacological properties, and identified signals.

#### 4.6.3 Actionable Recommendations

Better estimation of the duration of pregnancy safety studies is essential for planning. Defining a target sample size upfront helps determine feasible study design approaches (e.g., active prospective registry, passive surveillance studies, linked electronic healthcare claims and/or EMR data). Clear sample size targets for priority outcomes (refer to Section 4.5) and appropriate follow-up duration is essential to better estimate study duration and optimize planning efforts.

Estimating pregnancy exposure for new products might be challenging, especially in case of inadvertent exposure. Extending a study with insufficient recruitment or unmet objectives may not be appropriate. If recruitment goals are not met despite best efforts, adjustments may be necessary in consultation with regulatory authorities. It is therefore important for MAHs to discuss study progress with regulators at regular intervals, such as during the submission of annual study progress reports, to revise study plans when recruitment and retention fall below initial expectations. Stopping rules could be defined in the protocol for cases where the number of outcomes occurring in a pre-defined period is below the pre-defined threshold (in the context of adequate recruitment and follow-up).

### 4.7 Special circumstances: multi-stakeholder collaborations

#### 4.7.1 Overview of example studies

Of the example studies reviewed, only one is a collaborative study spanning multiple MAHs (Multiple manufacturers-COVID-19 vaccines; Table 5). This study is a primary data collection study of COVID-19 vaccines, covering more than 20 countries with a target sample size of at least 6,000 individuals (at least 500 per vaccine brand). Besides this study, the geographical representation of pregnancy registries was notably limited and most passive pregnancy surveillance studies predominantly consisted of cases from the US. The multi-product COVID-19 vaccine pregnancy registry stands out for its global recruitment efforts and innovative approach of engaging pregnant people through social media platforms, representing an advancement beyond traditional recruitment methods. The absence of multi-stakeholder collaborative studies among secondary data analysis studies likely reflects that, while studies may utilize common real-world data sources, it may be currently more efficient for each MAH to develop separate study protocols and analytic plans that generate product-specific results rather than collaborating in a pre-competitive scenario.

#### 4.7.2 Current guidance

In current guidance and recent workshops held to advance optimal approaches to generate high quality, timely safety data about product use during pregnancy, the FDA recommends collaborations across MAHs and within therapeutic areas. These collaborations would drive efficiency across stakeholders (i.e., patients/providers/MAHs/regulatory authorities), increase quality, and promote trust. The anti-retroviral therapy pregnancy registry (APR), a multi-sponsor study that has been ongoing for over 30 years to monitor antiretroviral (ARV) drug use during pregnancy for early signs of potential teratogenicity, is frequently cites as an exemplar of a successful cross-industry collaboration (Albano et al., 2024).

Recent EMA guidance does not specifically recommend cross-industry collaboration but it highlights two key advantages: (1) methodological benefits of evaluating the impact of product use during pregnancy through multi-product or disease-based registries, including the ability to construct internal comparators and better control of potential confounding due to indication, and (2) efficiency gains to overcome relatively low pregnancy exposure and outcomes prevalence through well-designed, large, multi-national/multi-database approaches that may utilize common data models or data pooling (e.g., Nordic registers).

#### 4.7.3 Actionable recommendations

Outside of exceptional examples like the APR and the North American Antiepileptic Drug (AED) Pregnancy Registry, which were set up with successful partnerships across MAHs with common post-marketing commitments/requirements to study drugs with expected intended use during pregnancy, there have been barriers for MAHs to collaborate on a single study or shared platform. These barriers to collaboration include differences across MAHs on timelines of approval/milestones, labeling of indication and treatment guidelines, variation in uptake and footprint, feedback from regulatory agencies that does not support a harmonized approach, lack of common data/operational infrastructure, funding structures, and confidentiality concerns about sensitive information.

Establishing an independent, common registry or RWD platform with a core protocol that is hosted by a third party, and from which MAHs with similar needs could partner for study conduct of secondary database studies, is a potential solution to overcome many of the current barriers to pre-competitive collaborative approaches. A key enabler for driving scientifically rigorous and efficient evidence generation would be the creation of a minimum core set of safety outcomes aligned across health authorities (Table 7) and vaccine MAH.

To facilitate and incentivize the creation of multi-sponsor registries that are fit for purpose to fulfil post-approval safety and/or effectiveness requirements, more guidance and harmonization across regulatory agencies is needed, including flexibility for registry platforms to be owned by third parties (e.g., contract research organizations). Compared to traditional, single product registries, the benefits of multi-sponsor registries can include efficiency in financial and human resource requirements (MAH, regulatory agency, investigators), time (from study start to delivery of actionable evidence), and participants number (simplified enrolment reducing burden, re-use of comparator/un-exposed groups). Through collective alignment on critical design and analytic specifications, multi-sponsor registries can drive scientific rigor to minimize bias and enhance reliability of results. Finally, their larger and more representative sample sizes will allow for reduced uncertainty and improved confidence in the study results. For vaccines without an expected recommendation for use during pregnancy, these benefits would be even more acute, since lower uptake rate would likely render traditional registry approaches unsuccessful.

### 4.8 Special circumstances: rapid cycle analyses

#### 4.8.1 Overview of example studies

In addition to the traditional cohort study designs that have been described thus far, other analysis techniques may also be used to generate safety data in pregnancy. For example, one study (Abrysvo-RSV; described in Table 6) utilizes both a rapid cycle analysis component and a subsequent cohort study analysis. Rapid cycle analysis is a technique to rapidly identify potential signals associated with vaccine safety (Klein et al., 2021; Yih et al., 2011). The technique uses the maximized sequential probability ratio test (maxSPRT) to conduct repeated statistical tests to determine whether there is a significant association between an exposure and an outcome. To account for the repeated testing procedure and reduce the rate of false positives, the proportion of total alpha that is used at each test is spent according to the proportion of total information that has been received at the time of the test (and by the predetermined shape of the alpha spending function that is selected). In contrast to a traditional cohort study in which sufficient sample size must be achieved before statistical testing can be initiated, the rapid cycle analysis allows for testing to begin as soon as data begins to accrue. While this technique can help to identify potential safety signals, confounding control is typically limited given small available sample size, and thus is best followed up with a well-designed and large cohort study when feasible to assess causality. The increasing use of rapid cycle analysis prior to conducting a well-designed cohort study allows for early identification of vaccine safety information, which can inform appropriate risk management.

There was no use of a rapid cycle analysis in the primary data collection studies identified.

#### 4.8.2 Current guidance

Despite an increasing need to generate faster safety evidence, no guidance was identified from the FDA or EMA regarding the use of rapid cycle analyses in post-approval pregnancy safety studies.

#### 4.8.3 Actionable recommendations

Additional guidance on the role of rapid cycle analyses for the generation of safety evidence is needed from regulatory authorities. This includes information on when a rapid cycle analysis might be most effective (e.g., for vaccines recommended vs. not recommended in pregnancy, outcomes) and additional information regarding basic requirements for this method to be implemented (e.g., recommended data sources).

## 5 Limitations

The following limitations should be considered when reviewing the conclusions from this work. First, only a small number of example studies were selected for review for demonstration purposes. Therefore, the selected studies may not be representative of the entire body of safety evidence generation work in vaccine pregnancy research. Second, not all studies had detailed protocols available, which limited what could be extracted about the planned study design (e.g., target sample size, outcomes collected etc.). For example, a study might have collected an outcome without reporting it in the manuscript. Publishing more comprehensive information in the form of supplementary material should be encouraged in order to ensure study replicability and enhance transparency in research methodology. It is important to note that many of the example studies were designed or completed prior to the implementation of current regulatory guidance. Therefore, any divergence from present standards should be interpreted as temporal precedence rather than methodological non-compliance.

## 6 Conclusion

Overall, wide variation in approaches to study the safety of vaccines in pregnancy were identified. In part, this is likely due to the differences in the vaccines themselves, as well as their indications and exposed populations. This article has highlighted some key areas in which additional guidance for the conduct of post-approval safety studies of vaccines in pregnancy would be beneficial (see Table 8 for a summary). While it is noted that it is not possible nor recommended to provide guidance that would cover every anticipated and unanticipated scenario, this manuscript aimed to highlight some specific key areas where further guidance, particularly for vaccine-specific research, is needed. This is important because the process of generating safety evidence is not complete upon product approval. Instead, critical safety information continues to be generated post-approval, particularly among populations such as pregnant individuals who may have had inadequate representation in clinical trials. Consolidating guidance across regulatory authorities and making standards clearer will help to both increase the speed at which post-approval data can begin to be collected and improve the inferences that can be drawn from the study findings. Ultimately, the efficient and meaningful conduct of post-approval studies of vaccine safety is critical not just for manufacturers of these products, but to all stakeholders, including regulators, prescribers, and the population for which the vaccines are indicated.

**TABLE 8:** Summary of actionable recommendations.

Summary of actionable recommendations
**Study type** Outline any vaccine-specific guidance for study design selection (i.e., pregnancy registry vs. electronic data source vs. passive surveillance study) Across both vaccine and medicine studies, further describe specific criteria for selecting passive surveillance studies vs. active pregnancy registries
**Comparators** Develop more comprehensive recommendations on the selection of preferred and appropriate comparators for vaccine safety studies
**Exposure Period** Advise on the critical data elements to characterize vaccine exposure Develop additional guidance on appropriate exposure windows for vaccines
**Outcomes** Provide (1) a comprehensive list of a subset of outcomes that all studies must evaluate across indications and, within this list of required outcomes, (2) a prioritization of which outcomes are most important and thus should drive calculations of sample size and study design Provide a decision framework for selecting other additional outcomes Advise on when and how long follow-up is needed Recommend appropriate strategies for assessing childhood outcomes (e.g., growth and development) Harmonize expectations regarding validation expectations across regulatory authorities Provide guidance on the use of machine learning techniques to refine case definitions
**Enrolment target/Sample size** Provide expectations regarding minimal detectable risk and target statistical power for priority outcomes
**Study duration & criteria to close a post-approval registry** Clarify sample size targets for priority outcomes and appropriate follow-up duration (including stopping rules)
**Special circumstances: multi-stakeholder collaborations** Agree on a minimum core set of safety outcomes (See Outcomes section) Provide more guidance and harmonization across regulatory agencies to facilitate and incentivize the creation of multi-sponsor registries, including flexibility for registry platforms to be owned by third parties
**Special circumstances: rapid cycle analyses** Provide guidance on the role of rapid cycle analyses for the generation of safety evidence, including when a rapid cycle analysis might be most effective and basic requirements for this method to be implemented

## Footnotes

1 Of note, guidance from the Brighton Collaboration (Munoz et al., 2015; Jones C. E. et al., 2016), while referenced in later sections, is not summarized in Table 1 because it is not specific to non-interventional studies, nor does it originate from a regulatory authority.

2 For example, the Advisory Committee on Immunization Practices (ACIP) in the US

3 Late pregnancy generally refers to the final weeks leading up to delivery, typically from 34 weeks of gestation to term (37–40 weeks), although the exact definition may vary depending on the clinical guidelines and practices in different countries

4 Varivax Registry initiated in 1995, complemented in 2006 by ProQuad, indicated for children aged 12 months through 12 years, and Zostavax, licensed for the prevention of herpes zoster among persons aged ≥50 years
